# 

**Published:** 1781-05

**Authors:** 


### SECTION IV.

### MONTHLY CATALOGUE.

I. *Hints on Diseases that are not cured; addressed to the Faculty only.* 4to. *Murray*, London, 1781. 46 pages. 1s. 6d.

THIS pamphlet is written in an agreeable manner, and seemingly with a view to draw the attention of medical practitioners to certain diseases usually deemed incurable, rather than to propose any new modes of treatment in such cases. The author has confined his reflections chiefly to consumption and gout. "At a period, " says he, which teems with valuable treatises, " on almost every other disease; and when we " are threatened with an inundation of simple " facts from every quarter, the number of vo- " lumes on pulmonary phthisis begins to dimi- " nish, and the gouty patient is left to tell his " own story."

As a means of improving our practice in cases of phthisis, he proposes the institution of an infirmary, which might consist of one or more houses, in a dry, airy, but not very exposed situation, for the reception of poor consumptive

ſumptive patients. " If one of them, he ob-

" ſerves, were ſituated near the ſea, it would

" afford the probable advantages of ſea air, and

" an opportunity of ſailing, an exerciſe ſo highly

" extolled, that we have ſome reaſon to think

" on this confined ſcale, it might prove no deſ-

" picable auxiliary. The relative ſituation of a

" lime-kiln, or any manufactory which occa-

" ſions copious exhalations, may determine the

" choice of other receptacles. Let each of

" them be ſpacious; yet never contain more

" patients than one phyſician and one apothe-

" cary may well take the charge of: the for-

" mer ſhould attend at leaſt three times a week,

" and keep an exact regiſter of every caſe that

" falls under his inſpection. His ſagacity muſt

" direct him further; but he will, I believe,

" ſee the propriety of proceeding on the plan

" of ſimple, and cautious medicinal treatment

" already recommended. He will firſt apply

" himſelf to learn the beſt regimen, and how

" far it alone may be relied on; and when he

" ſhall have occaſion to call in remedies to his

" aid, he will give them one by one a fair trial,

" and thus he will be able to aſſign to each its

" reſpective degree of merit."

In

In gouty cafes he propofes, that the books which make mention of anti-arthritic medicines fhould be confulted, and that all fuch as are not abfurd or evidently infignificant, fhould have a fair trial, and their effects be carefully attended to.

2. Outlines of the theory and cure of fever, upon plain and rational principles, by *John Aitken*, Fellow of the Royal College of Surgeons, of the Royal Medical Society, one of the Surgeons of the Royal Infirmary, and lecturer on anatomy, furgery, and chemiftry in Edinburgh, 8vo. *Cadell*, London, 1781. 88 pages.

The firft thing that attracted our notice, on opening this little production, was the following dedication to COMMON SENSE: "Thou venerable arbiter of fcience and of human conduct, "univerfally to be cultivated and adored! How "feldom do the fons of Æfculapius bend as "humble votaries at thy fhrine! Many of them "have lamentably fecluded themfelves from thy "irradiating influence; fome bewildered by "numberlefs mounds of mud, their own creation; fome confounded by artificial clouds "and darknefs; and fome, ftrange to tell! have "fatally deviated from the path leading to thy "temple, becaufe it is patent and obvious. Of-

"tener

"tener have they idolatrously become the steady "devotees of chaos, thy potent and gloomy "rival, bending to the fictions of their mon- "strously teeming imaginations.

"If ever thy feeblest ray has in any degree "illumined my mind, deign to accept this dedi- "cation of *Outlines of the Theory and Cure of* "*Fever*, as a respectful oblation from thy hum- "blest admirer, THE AUTHOR."

Mr. Aitken supposes a *plastic power* to be inherent in animal as well as vegetable bodies, the operation of which he names *the plastic or healing process*, because it tends directly to the cure of disease. He thinks that the art of physic possesses little or no immediate influence over this principle, and is only capable of assisting or co-operating indirectly with it. He rejects the use of blisters in the cure of fever, because "they "are manifestly deranging powers, and therefore "ill suited to promote or second the plastic "process."

3. Elements of anatomy and the animal oeconomy. The second edition, with considerable alterations and additions. By *Samuel Foart Simmons*, M. D. F. R. S. &c. 8vo. *Walker*, London, 1781. 418 pages. 5s. *in boards.*

With

With the firſt edition of this uſeful compendium, our anatomical readers are doubtleſs well acquainted. In the preſent edition it is greatly enlarged and improved. Amongſt other additions Dr. Simmons has given a table of the muſcles, deſcribing the origin, inſertion, and uſe of each; he has likewiſe introduced a more copious deſcription of the brain than is to be met with in the firſt impreſſion, and as he has availed himſelf of all the lateſt improvements in anatomy, his work cannot but prove highly acceptable to medical ſtudents.

4. A treatiſe on the gonorrhœa; to which is added a critical enquiry into the different methods of adminiſtering mercury. Intended as a ſupplement to a former work, intitled, A new and eaſy method of cure by the introduction of mercury into the ſyſtem through the orifices of the abſorbent veſſels on the inſide of the mouth. By *Peter Clare*, ſurgeon, ſmall 8vo. *Cadell*, London, 1781. 60 pages.

The greateſt part of this pamphlet conſiſts of quotations from Boerhaave, Mead, Woodward, Alſton, Geo. Fordyce, Smith, and Plenk, but chiefly from the three latter. Mr. Clare avows himſelf a convert to the uſe of cold vitriolic injections in gonorrhœa, provided at the ſame time

ſame time a grain of mercurial powder is daily applied to the cuticular ſurface of the lips. Mercurial preparations he has generally found too irritating. His critical inquiry ſeems intended to prove, that the rubbing calomel on the inſide of the mouth is the ſafeſt and moſt effectual method of introducing mercury into the ſyſtem.

5. Recherches analytiques ſur les eaux de la fontaine minerale de Jaleyrac dans la Haute Auvergne, avec un precis des maladies où ces eaux peuvent être utiles, des cas où elles pourroient être prejudiciables, et la maniere de les prendre avec ſuccés. Par M. *Pierre André de la Rouſſerie*, D. M. Medecin a Neuvie en Limouſin. *i. e.* Analytical inquiries concerning the mineral waters at Jaleyrac in Upper Auvergne, with an account of the diſeaſes in which they may be uſeful, of the caſes in which they might prove hurtful, and of the method of taking them with ſucceſs. By *Peter Andrew de la Rouſſerie*, M. D. Phyſician at Neuvie in Limouſin. 12mo. Tulle. 1780. 21 pages.

6. Diſſertation ſur le charbon malin de la Bourgogne, où la puſtule maligne : ouvrage couronné par l'academie des ſciences, arts & belles lettres de Dijon, le 14 Fevrier, 1780,

*i. e.* A diſſertation on the carbuncle or malignant puſtule of Burgundy, being the work crowned by the academy of ſciences, arts, and belles lettres at Dijon, Feb. 14, 1780. 8vo. Dijon, 1780.

7. Compendio di notizie intereſſanti circa il veneno di rabbioſi animali. *i. e.* A compendium of intereſting remarks on the poiſon of mad animals. By *Felix Aſti*, M. D. 4to. Mantua, 1779, 110 pages.

This compilation would have been more uſeful, if the author had availed himſelf of the reſearches of ſeveral modern writers, with whoſe works he ſeems to be unacquainted. Towards the cloſe of the pamphlet we meet with a few caſes, from which we learn, that ſeveral perſons at Pomponeſco ate part of a hog that had been bit by a mad dog, without experiencing any ill effects from it. The author likewiſe informs us, that the fleſh of an ox under ſimilar circumſtances proved equally innocent. He ſpeaks of a mad wolf that bit a great number of perſons in the neighbourhood of Lonato, moſt of whom periſhed. One woman, however, was cured by the internal and external uſe of mercury; and another, who was delivered of a child the day after the accident, eſcaped by taking Bellini's powder, powder, which is a mixture of cantharides and pepper. It does not appear, however, that either of these two patients had received the infection.

8. Eloge de M. Maret, Maitre en Chirurgie a Dijon, ancien chirurgien major de l'hospital general, pensionnaire veteran de l'academie des sciences, arts et belles lettres de la meme ville, par *M. Maret*, Docteur en Medecine, secretaire perpetuel de l'academie: lû dans la seance du 17 Decembre 1780. *i. e.* The Elogy of M. Maret, surgeon at Dijon, formerly surgeon to the General Hospital, and member of the academy of sciences, &c. by *M. Maret*, M. D. secretary to the academy. Read at the meeting on the 17th of December 1780. 8vo. Dijon. 32 pages.

John Philibert Maret, the subject of this little work, was born at Dijon, November 8, 1705. He studied surgery first under his father at Dijon, and afterwards at Rome and Paris. He died October the 14th 1780, unmarried, leaving the author and seven other nephews and nieces to regret his loss. He was the author of three dissertations presented to the academy of Dijon. In one of these he attempts to prove, that in dying persons hearing is the last fenfe extinguiſhed. His ſecond paper was on amputation, and the third on lithotomy.

9. Detail des ſuccés de l'etabliſſement que la ville de Paris a fait en faveur des perſonnes noyées, et qui a été adopté dans diverſes provinces de France. Par *M. Pia*, ancien echevin de la ville de Paris, & chevalier de l'ordre du roi, &c. *i. e.* An account of the ſuccefs of an eſtabliſhment inſtituted by the city of Paris in favour of drowned perſons, and which has been adopted in ſeveral provinces of France. By *M. Pia*, formerly ſheriff of the city of Paris, and knight of the king's order. 8vo. Paris. 3d edition, 1780.

10. Phyſica hominis ſani, ſeu explicatio functionum corporis humani, auctore *Nicolao Jadelot* regis conſiliario et medico, anatomiæ & phyſiologiæ in univerſitate Nanceiana profeſſore, academiæ regiæ ſcientiarum et artium Nanceianæ ac ſocietatis regiæ medicæ Pariſinæ ſocio; noſocomii Nanceiani a Sancto Carolo dicti medico. 8vo. Nanceii, 1779, 249 pages.

An inaccurate compendium of phyſiology, written in an obſcure ſtyle.

11. *Alberti von Haller* primæ lineæ phyſiologiæ auctæ ab *H. A. Wriſberg*, M. D. anatomes et et artis obftetric. prof. ordin. Gotting. 8vo. Göttingen, 1780, 550 pages.

This excellent work is greatly improved by the prefent editor's judicious notes.

12. Einleitung in die Wiffenfchaft der rohen und einfachen Arzneymittel, &c. Von *J. G. Gleditfch*, D. A. D. &c. *i. e.* An introduction to the knowledge of fimple medicines upon phyfico-chemical and medico-practical principles. By *J. G. Gleditfch*, M. D. and profeffor of botany at Berlin. 8vo. Berlin, 1779, 2 vol.

In this work the medicinal fimples are defcribed with great precifion, and the characteriftics of their goodnefs or adulteration carefully afcertained.

13. *J. F. Van Berkley's* Naturgefchichte von Holland, &c. *i. e.* The natural hiftory of Holland, by *J. F. Van Berkley*. Tranflated from the Dutch. With copper plates. Vol. I. 8vo. Leipfic, 1779, 314 pages.

This writer appears to be unacquainted with many late difcoveries in natural hiftory. He confines himfelf chiefly to a defcription of the atmofphere, foil, rivers, &c. of Holland, and paffes flightly over its animals, vegetables, and minerals. In his arrangement he follows M. M. Buffon and D'Aubenton.

14. Theoriæ

14. Theoriæ phænomenorum electricorum, quæ, seu electricitatis ex redundante corpore in deficiens trajectu, quæ sola atmosphæræ electricæ actione gignuntur. Auctore *J. de Herbert*, prof. philosophiæ naturalis. 8vo. Vienna, 1779, 246 pages, with copper plates.

This very ingenious performance, contains a great number of new and interesting experiments in electricity. In his seventh chapter, speaking of the effects of electricity upon organized bodies, the author mentions an experiment on two boys by weighing them in a pair of scales, one of which was suspended by silk; and he assures us, that the boy in the latter scale, after being strongly electrified, became much lighter than the other. Having procured recent portions of a nerve, muscle and artery, he found the first to be as good a conductor as metal, while the muscle was less so, and the artery did not conduct at all. But the next day the nerve, when dry, was as incapable of transmitting electricity, as the artery. Hence our author concludes, that its conducting powers depended on the existence of a nervous fluid. In noticing the medicinal effects of electricity, our author tells us he has often observed, that after the cure of obstinate diseases of long standing,

ſtanding, ſuch as palſies, for inſtance, by electricity, the removal of the original complaint has been ſucceeded by rheumatic fevers, catarrhs and diarrhœas.

15. Abhandlungen zur naturgeſchichte, phyſik und oekonomie, aus den Philoſophiſchen Tranſactionen und Samlungen, vom erſten Bande angefangen geſamelt und mit einigen Anmerkungen überſetzt erſter Band. *i. e.* Eſſays on natural hiſtory, natural philoſophy, and rural œconomy, extracted from the Philoſophical Tranſactions, with notes. Vol. I. 4to. Leipſic, 1779, 292 pages.

The German reader is indebted for this uſeful compilation to Mr. Leſke, profeſſor of natural hiſtory at Leipſic, who has enriched it with a great number of uſeful notes.

16. *H. A. Wriſberg*, M. D. et anatomes profeſſoris diſſertatio de præternaturali et raro inteſtini recti cum veſica urinaria coalitu et inde pendente ani defectu obſervationibus anatomicis ſuperſtructa. Cum figuris. 4to. Göttingen, 1779.

The ſubject of this diſſertation was a child, who died eight days after its birth. The appearances on diſſection are well exhibited in the engravings that accompany the work.

17. *H. A. Wriſberg*, M. D. &c. Obſervationes anatomicæ de teſticulorum ex abdomine in ſcrotum deſcenſu, ad illuſtrandam in chirurgiâ de herniis congenitis utriuſque ſexus doctrinam, cum figuris. 4to. Göttingen, 1779.

The intention of our author is to ſhew, how the deſcent of the teſticles may give riſe to an hernia congenita. He firſt quotes the obſervations of different modern anatomical writers, and then adds the reſult of his own inquiries on this ſubject. He finds, that the teſtes do not deſcend ſo low as the abdominal rings till the ſixth or ſeventh month, and that the ſcrotum till that time is ſmall and empty. He obſerves, that the teſticles while in the abdomen are covered only by a minute proceſs of the peritonæum, and afford no appearance of a tunica vaginalis. In his diſſections, he has ſeveral times found a part of the omentum or inteſtines adhering to the teſticle in the abdomen of the fœtus, and when this happens, an hernia congenita will be likely to take place. The ſame thing, he adds, will occur, when the peritonæum in its courſe over the ſeminal veſſels to the meſentery, ſends off a minute proceſs to the ileum or cæcum, and by means of it draws down the inteſtines on the right ſide, which is the common ſeat of the hernia congenita.

